# The basic reproduction number and prediction of the epidemic size of the novel coronavirus (COVID-19) in Shahroud, Iran

**DOI:** 10.1017/S0950268820001247

**Published:** 2020-06-10

**Authors:** A. Khosravi, R. Chaman, M. Rohani-Rasaf, F. Zare, S. Mehravaran, M. H. Emamian

**Affiliations:** 1 Ophthalmic Epidemiology Research Center, Shahroud University of Medical Sciences, Shahroud, Iran; 2 Department of Epidemiology, School of Public Health, Shahroud University of Medical Sciences, Shahroud, Iran; 3 Center for Health Related Social and Behavioral Sciences Research, Shahroud University of Medical Sciences, Shahroud, Iran; 4 ASCEND Center for Biomedical Research, Morgan State University, Baltimore, USA

**Keywords:** COVID-19, epidemic, incident case, Iran, projection, reproduction number

## Abstract

The aim of this study was to estimate the basic reproduction number (*R*_0_) of COVID-19 in the early stage of the epidemic and predict the expected number of new cases in Shahroud in Northeastern Iran. The *R*_0_ of COVID-19 was estimated using the serial interval distribution and the number of incidence cases. The 30-day probable incidence and cumulative incidence were predicted using the assumption that daily incidence follows a Poisson distribution determined by daily infectiousness. Data analysis was done using ‘earlyR’ and ‘projections’ packages in R software. The maximum-likelihood value of *R*_0_ was 2.7 (95% confidence interval (CI): 2.1−3.4) for the COVID-19 epidemic in the early 14 days and decreased to 1.13 (95% CI 1.03–1.25) by the end of day 42. The expected average number of new cases in Shahroud was 9.0 ± 3.8 cases/day, which means an estimated total of 271 (95% CI: 178–383) new cases for the period between 02 April to 03 May 2020. By day 67 (27 April), the effective reproduction number (*R*_t_), which had a descending trend and was around 1, reduced to 0.70. Based on the *R*_t_ for the last 21 days (days 46–67 of the epidemic), the prediction for 27 April to 26 May is a mean daily cases of 2.9 ± 2.0 with 87 (48–136) new cases. In order to maintain *R* below 1, we strongly recommend enforcing and continuing the current preventive measures, restricting travel and providing screening tests for a larger proportion of the population.

## Introduction

According to the World Health Organization COVID-19 dashboard [1, 2], as of 29 April 2020, the novel coronavirus pandemic has spread to 213 countries, areas or territories with 2 959 929 confirmed cases and 202 733 confirmed deaths. Some resources even report higher numbers of patients and deaths, and the numbers continue to escalate. [3] The Islamic Republic of Iran was the first Middle East country to report a case of death (19 February 2020) and is currently among countries with high prevalence of COVID-19 [1, 3]. By 29 April, there were 92 584 confirmed cases in Iran, 5877 of which had deceased [3]. Given the rapid spread of the virus, the government immediately responded by establishing more than 40 laboratories within two weeks after the start of the epidemic, and consequently, there was a sudden spike in the reported number of positive cases. Currently, there are 126 laboratories actively conducting PCR testing throughout the country.

**Fig. 1.**
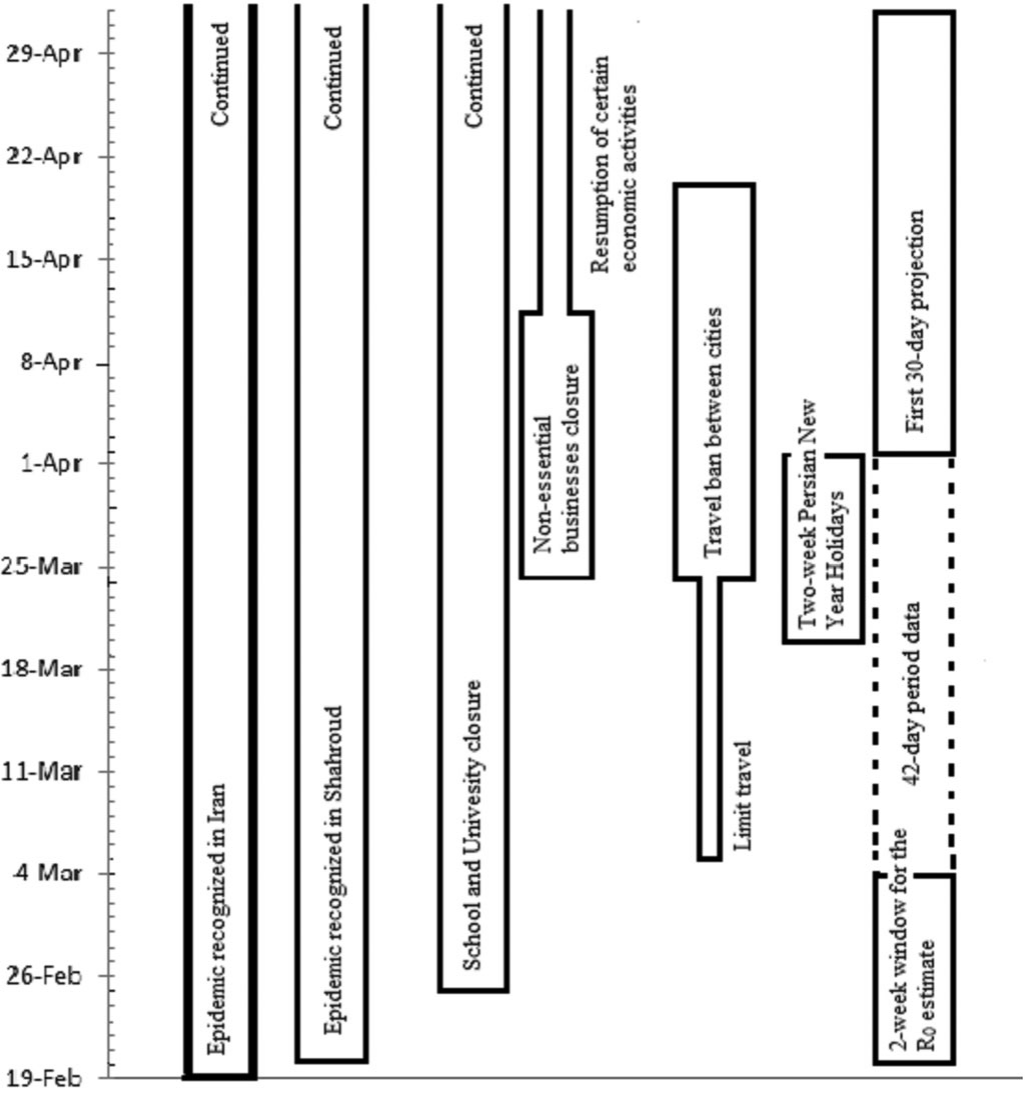
The timeline of COVID-19 epidemic in Shahroud, Iran, 2020.

The timeline graph of the epidemic is presented in Figure 1. The first cases were immediately reported to the Health Department, and preventive protocols were developed and put in place to limit the further spread of the infection. These included cancelling in-person classes in schools and universities as of 25 February 2020, and switching to online platforms, as well as public awareness campaigns that encourage citizens to minimise face-to-face contact and promote social distancing. Since the timeframe from 20 March to 02 April coincides with the Persian New Year or ‘Norouz’ holidays in Iran, there were even more stringent restrictions to limit the social activities, visiting family and friends, trips, shopping and festivals that are considerably more common around this time of the year.

In epidemics, close monitoring and evaluation is necessary to investigate the efficiency of control measures, determine the potential community transmission patterns and predict the progression of the epidemic and the trajectory of the epidemic curve. One useful epidemic measure that helps investigate the transmissibility of infection is the reproduction number (*R*). The basic reproduction number (*R*_0_) is the average expected number of new cases infected by a primary case and must be estimated early during an epidemic [4, 5]. *R*_0_ can be affected by various factors such as the probability of transmission upon contact between an infected case and a susceptible person, the frequency of contact and the duration of infection in a person [6]. As the epidemic progresses, when the population begins to become immune and/or interventions are put in place, the effective reproduction number (*R*_t_) can be estimated in real time [7]. The serial interval (SI) of an infection is the mean duration between the symptom onset of two successive cases (the primary case and the secondary case). The force of infection (denoted *λ*), which describes the rate at which susceptible people acquire a given infection, is another useful parameter when implementing preventative measures [8].

**Fig. 2.**
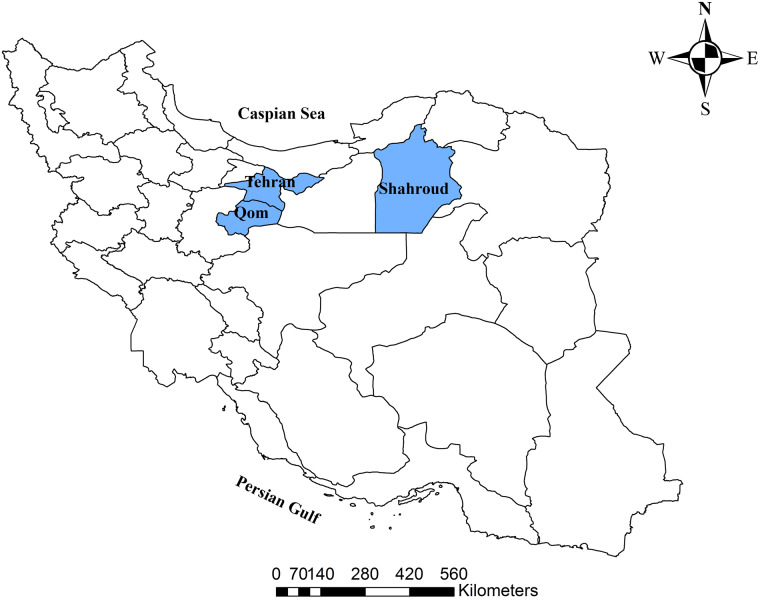
Map of Iran showing the locations of Shahroud County and Qom and Tehran Provinces. The first case of COVID-19 was identified in Qom which by road is located 550 km away from Shahroud.

According to the unpublished report of the Ministry of Health and Medical Education in Iran, the incidence rate of COVID-19 has been highest in Semnan Province (118 cases per 100 000 persons) and the highest incidence rate in Iran by 01 April was seen in Shahroud County. Shahroud, in Shahroud County and Semnan Province, is a city located in Northeastern Iran with a population of about 218 628 in 2016 [9]. The first confirmed case of COVID-19 in Iran was identified on 19 February in Qom which is about 550 km from Shahroud (Fig. 2). Four days later (23 February 2020), nasopharyngeal and throat swabs of five suspected cases in Shahroud were submitted for viral nucleic acid testing, and two tested positive. One of these primary cases was a 74-year-old woman who had been hospitalised on 10 February, with chief complaints of fever and cough, and a travel history to Qom. This indicates that the epidemic probably started almost one month before it was known to the public. Given the high incidence rate in the region, the aim of this report is to estimate the *R*_0_ of the COVID-19 in the early stage of the epidemic (20 February to 04 March) and predict the trajectory of the epidemic and new cases in Shahroud.

## Methods

The protocol of this study was reviewed and approved by the Institutional Review Board of Shahroud University of Medical Science (IR.SHMU.REC.1398.160). The data for this study were all confirmed cases of COVID-19 in Shahroud County. The majority of cases were patients who were referred to Imam Hossein hospital (which is currently the only specialty hospital designated for the management of COVID-19 patients in Shahroud County); others were positive cases from public health centres. In the first month of the epidemic, all walk-in and referral cases were screened. Of these, suspected cases were admitted and tested. After establishing new testing centres, the protocol was revised on 21 March 2020 so that all suspected outpatients would be tested at a public health care setting. For testing, two respiratory tract samples (throat and nasopharyngeal swabs) are collected and submitted for viral nucleic acid testing. All positive cases are systematically recorded in a designated registry which is used for follow-up and contact tracing.

In this study, we used an informative prior distribution for the SI, which was estimated as 7.5 ± 3.4 days for COVID-19 in Wuhan, China [10], fit with a gamma distribution. We calculated the likelihood-based *R*_0_ using a branching process with Poisson likelihood. Bootstrapping with 1000 times resampling was used for obtaining the distribution and confidence interval (CI) of *R*_0._

We then used the estimates of *R*_0_, SI and daily incidence to simulate the trajectories and project the future daily cumulative incidence where the main assumption was that the model follows a Poisson distribution [5]. For each date *t* ≥ 2, the number of incident cases *I*_*t*_ was drawn from a Poisson distribution with mean 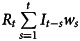, where *R*_*t*_is the instantaneous reproduction number, *w*_*s*_ is the discrete SI distribution and *I*_*t*−*s*_ is the incidence at time step *t* – *s*.

For a 30-day projection, we used a uniform distribution of 0.8−1.3 for *R*_0_ and bootstrapping with 1000 times resampling [5, 11]. For the projection in the next 30 days, we estimated a *R*_0_ of 0.8−1.3 based on the cultural background and the public health information level of the community, as well as the extent of case finding strategies.

We also estimated *R*_t_ for the first 67 days of the epidemic using the approach proposed by Cori *et al*. [7]. Data analysis was performed in Microsoft Excel and R (3.6.3) software using the ‘incidence’, ‘earlyR’, ‘ggplot2’ and ‘projections’ packages.

## Results

### *R*_0_ estimation for 20 February to 01 April 2020

**Fig. 3.**
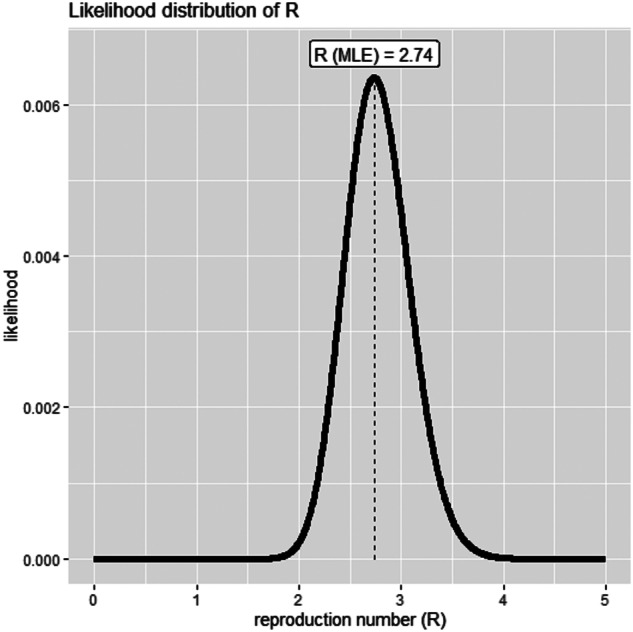
The distribution of likely values of basic reproduction number (*R*_0_) with the maximum-likelihood estimation for the beginning of the epidemic (20 February and 04 March 2020).

Using the SI distribution for the first two weeks (20 February to 04 March 2020), the maximum-likelihood value of *R*_0_ was estimated at 2.7 (95% CI 2.1–3.4) for day 14 (04 March 2020), which is indicative of a propagated epidemic (Fig. 3). The maximum-likelihood value of *R*_0_ decreased to 1.28 (95% CI 1.14–1.43) for day 30 (20 March 2020) and 1.13 (95% CI 1.03–1.25) for day 42 (01 April 2020).

### Actual number of cases from 20 February to 26 April 2020

During the first 42 days of the epidemic (20 February to 01 April 2020), a total of 993 suspected samples were tested for COVID-19 in Shahroud, and 424 (42.7%) of them tested positive. Among the positive cases, 47 people (11.1%) were public health cases or outpatients. During the timeframe between 02 April and 26 April, a total of 1208 new suspected cases were tested for COVID-19 and 114 new confirmed cases (4.6 cases/day) were recorded during this period.

**Fig. 4.**
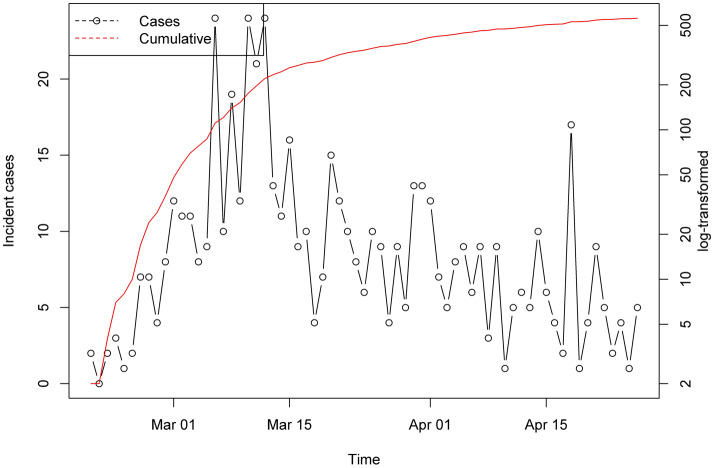
Incidence and cumulative incidence of COVID-19 between 20 February and 26 April 2020 in Shahroud, Iran.

The daily observed distribution of these confirmed cases by 26 April is illustrated in Figure 4.

### First 30-day projection for the period between 02 April to 03 May

**Fig. 5.**
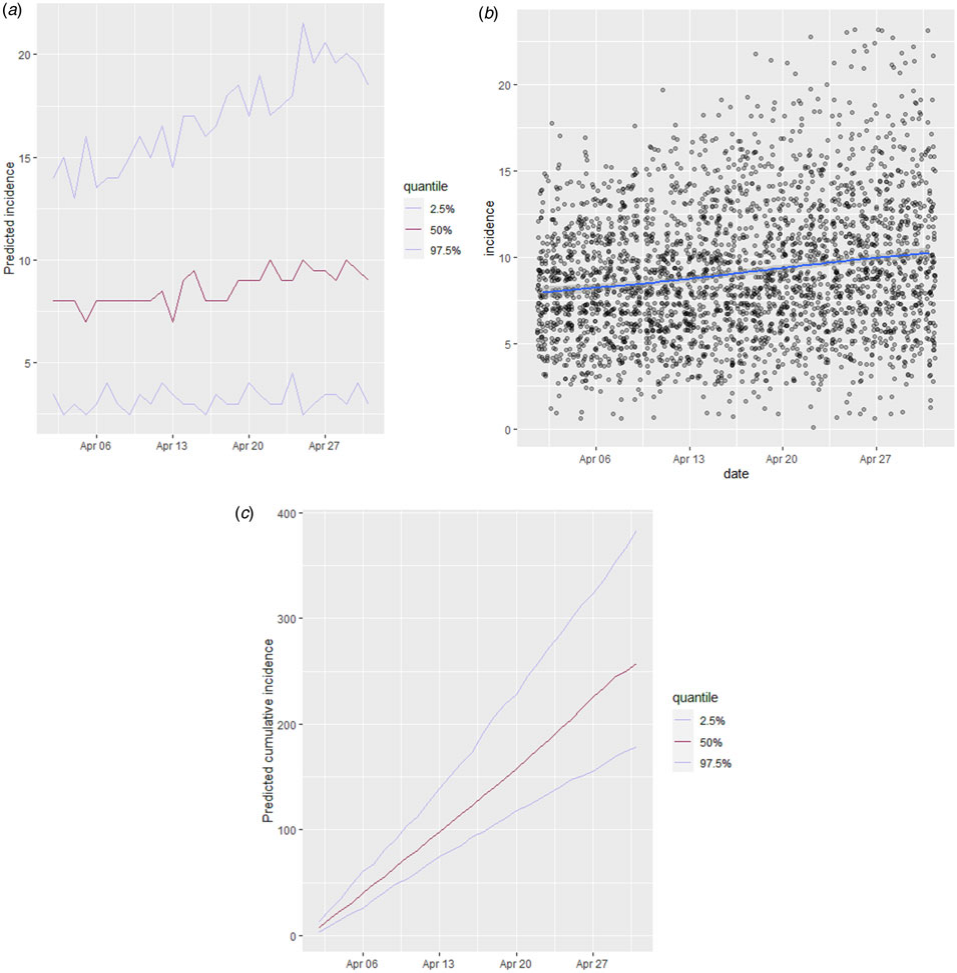
Thirty-day projections of the incidence and cumulative incidence of COVID-19 in Shahroud, Iran. (a) Predicted number of incident cases, (b) smoothed number of predicted cases, (c) cumulative incidence if the basic reproduction number follows a uniform distribution of 0.8−1.3.

The predicted number of new cases for days 43−73 of epidemic (one month after the Persian New Year holidays), based on decreasing function of *R*_0_ between 1.3 and 0.8, is demonstrated in Figures 5a–c. The overall predicted average number of new cases was 9.0 ± 3.8 cases per day. In Figure 5b, the daily average of predicted incident cases was smoothed for the time span. The 30-day projected cumulative incidence in Shahroud is shown in Figure 5c; approximately 271 (95% CI 178–383) new cases were estimated to be infected.

### Second 30-day projection from day 68 (27 April to 26 May)

**Fig. 6.**
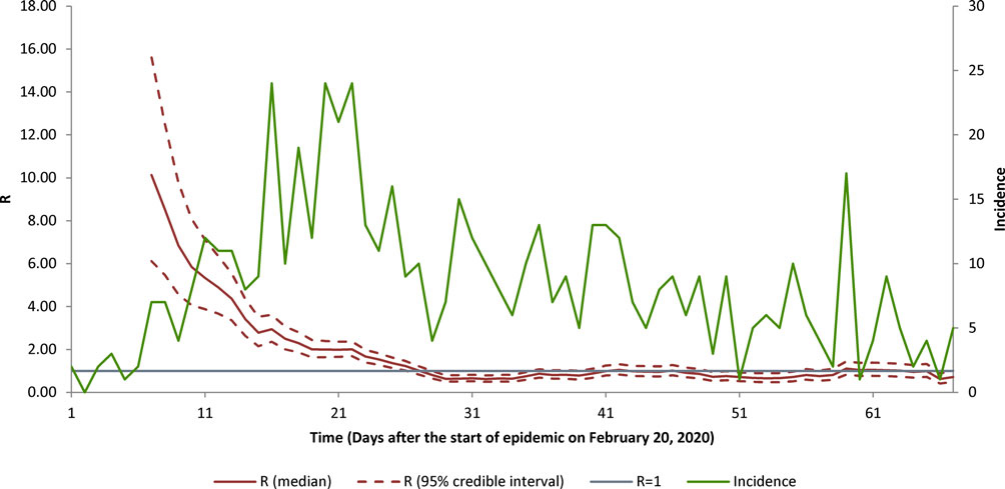
The instantaneous reproduction number and incidence number of COVID-19 for the first 64 days of the epidemic, Shahroud, Iran, 2020.

Based on the *R*_t_ for the last 21 days (days 46–67 of the epidemic), it is predicted that the mean daily cases for 27 April to 26 May 2020 will be 2.9 ± 2.0 with 87 (48–136) new cases. The *R*_t_ also showed a decreasing pattern to below 1 since day 28 of the epidemic which has remained near 1 to day 67 where it reaches 0.70 (Fig. 6).

## Discussion

The *R*_0_ of an infection is commonly used to characterise its transmissibility during an epidemic. The trend of *R*_0_ over time provides a measure of the effectiveness of control and prevention strategies in the community, and to control an epidemic, the goal is to reduce and keep the reproduction numbers below the value of 1 [7]. The basic reproduction number (*R*_0_) is the average number of secondary infections transmitted by each case in a susceptible population. The biological potential of an agent affects *R*_0_, but it also depends on contact rate in the population and duration of infectiousness. It can be used for projection and modelling of infectious disease spreading in the population. In this study, the estimated *R*_0_ of 2.7 (95% CI 2.1–3.4) during the early stage of the epidemic is in line with previous estimates [10, 12–14]. However, higher estimates of *R*_0_ have been reported in larger populations [15, 16]. For precise estimation of *R*_0_, certain conditions must be met which include the precise detection of cases in the early stages of the epidemic, restricting the calculation to a small timeframe [11], and choosing the appropriate estimation method [7, 17]. Since the number and patterns of people's contacts vary by country for cultural and educational reasons, the *R*_0_ can be different as well. Also, *R*_0_ can be different in sub-populations [18]. For precise detection of cases, all suspected cases (according to the screening protocol) and cases who have had close contact with confirmed cases should undergo viral nucleic acid testing. In Shahroud, there were 993 suspected cases, and 427 of them tested positive during the first 42 days of epidemic. However, in the early stages of the epidemic in Iran, there was limited capacity for testing, and the calculated *R*_0_ may be an underestimation. During the study period of 67 days, the number of tests performed during the first 42 days and the second 25 days was 926 (43% tested positive) and 1208 (9.4% tested positive), respectively, which indicates a considerable expanding of testing to the community. While increased testing and case finding is expected to be associated with higher *R*_0_ projections, we observed a steady decrease in *R*_0_ and *R*_t_ which can be attributed to the success of the interventions and not the higher number of immune persons. The mitigation and suppression strategies have managed to control the spread of the infection and reduce the reproduction rate through reduced contact and lower likelihood of transmission. Maintaining these strategies is expected to control the size of the epidemic and keep the *R* below 1.

The results of this study showed that *R*_0_ has decreased temporally. This pattern, which is promising for controlling an epidemic, is due to interventions enforced by the health system starting from the early days of the epidemic. Some of the most important measures were public education to promote social distancing and encouraging people to stay home. In addition, two other basic measures were taken in Shahroud: (1) At the time of hospital discharge, all patients and their caregivers were provided with counselling and training on how to be isolated at home for 14 days, and families received information about how to care for patients; (2) active contact tracing with follow-up of patients' family members and friends, work colleagues and other possible contacts and referral of suspected cases to medical centres [8].

According to our 30-day projection for 27 April to 26 May, there should be a decrease in *R*_0_, and a total of 87 new cases are expected. This can be due to the spread of the virus by unidentified asymptomatic cases and increased testing on outpatients. So, we strongly recommend measures to identify these cases for total control of the epidemic.

This study can inform health policymakers of the success of the preventive measures and interventions. It also emphasises the need for these measures to be continued along with stricter limitations in transportation until the transmission chain is broken and the epidemic is successfully controlled.

The main strengths of this study include its careful design, taking throat and nasopharyngeal swabs for testing of all suspected cases, and systematic recording of positive cases. One limitation was that testing in the first period was only done for those admitted in the hospital. There were also potential limitations in the calculation of *R*_0_. Given that *R*_0_ is the average of *R*_0i_ in population subgroups; its value may be higher in some high-risk subgroups. As a result, the epidemic could be ongoing in these subgroups [18]. As the number of immune persons increases in the community, we determined *R*_t_ which has decreased below *R*_0_.

In conclusion, the *R*_0_ of COVID-19 in Shahroud was considerably high at the onset of the epidemic, but with preventive measures and public education, it was reduced to 1.13 (95% CI 1.03–1.25) within 42 days. This reduction highlights the success of preventive measures in place, but mitigations and expansion of case finding should be continued. We strongly recommend mass screening and testing of suspected cases, travel restrictions especially during upcoming holidays and stringent monitoring of close contacts. These strategies will be challenged by the social and economic burden on the community, and sooner or later restrictions have to be relaxed. For example, low-risk business activities restarted in mid-April in most of the country, except Tehran. Our prescription for the community is to stop the traditional kiss, hug or hand-shake greeting gestures and place emphasis on face masks, hand hygiene, social distancing and strict monitoring of economic centres such as markets and public places.
